# Assessment of urban wetlands loss and fragmentation using land use and land cover change and guidostoolbox: A study of Chorrillos district, Lima

**DOI:** 10.1371/journal.pone.0314163

**Published:** 2024-12-04

**Authors:** Sally Torres Mallma, Anna Torres Mallma

**Affiliations:** 1 Facultad de Arquitectura y Urbanismo, Universidad Ricardo Palma, Lima, Peru; 2 Hispanic and Italian Studies, University of Illinois Chicago, Chicago, Illinois, United States of America; Van Lang University: Truong Dai hoc Van Lang, VIET NAM

## Abstract

The coastal landscape of the Chorrillos district is an ecosystem characterized by hosting the largest population of migratory and resident bird species in Lima, including the Protected Natural Area of the Pantanos de Villa Wildlife Refuge (RVSPV). In response to wetland loss, this study aims to explore the process of natural landscape fragmentation in the Chorrillos district, including the RVSPV, from 1985 to 2021 to identify the impact of anthropogenic agents on structural connectivity. The methodology involves (i) an analysis of land cover and land use (LULC) using the MapBiomas Peru Collection 1.0 Platform, (ii) a patch accounting analysis, (iii) an analysis of Area Density in the Foreground (FAD), and (iv) an analysis of Morphological Spatial Patterns (MSPA) using the GuidosToolbox (Graphic User Interface for Image Object Description and Shapes—GTB) Version 3.304. The results indicate a hierarchy of disturbed patches, with the largest patch (RVSPV) displaying specific spatial processes that contribute to landscape transformation and persistence. The ecological function of the patches and habitat links demonstrate the benefits of connectivity and environmental exchange between fragments, aiming to counteract landscape loss.

## 1. Introduction

According to the Convention on Wetlands (Ramsar), valuing the existence of wetlands means recognizing them as vital ecosystems for biological diversity on the planet and protecting them to ensure the continuity of their invaluable ecological functions. However, the continuous expansion of urban areas represents a significant threat to wetlands within and around metropolises [1]. Decades of research have revealed that wetlands have decreased by 35% worldwide between 1970 and 2015. Among all regions, Latin America leads this trend with a 59% loss in recent decades [2]. This urban growth in Latin American cities has negatively impacted coastal areas, leading to the degradation and marginalization of crucial natural ecosystems [1]. Changes in land use, such as urbanization, drainage, and agricultural expansion, are the primary causes of this loss, significantly affecting notoriously the ecosystem services wetlands provide [3,4].

As various studies reveal, critical study points for wetland conservation should not only focus on hydrological processes, biogeochemical cycles, etc. Still, they should also include analyzing one of the most critical wetland patterns: fragmentation. Some studies address habitat fragmentation, defined as a loss of physical continuity between different parts of the habitat or a dynamic process of alteration in the landscape resulting from its transformation into “patches" [5]. This loss involves identifying processes of spatial landscape transformation such as attrition, creation, dissection, fragmentation, perforation, and shrinkage [6], which allows us to distinguish different forms, sizes, and quantities of patches and their interrelationships. From the perspective of landscape ecology, spatial changes and the characterization of patches within the habitat are some variables that allow us to explore the spatial heterogeneity of the wetland, which contributes to the conservation and maintenance of ecological stability [7,8]. Following this, our perception of natural landscapes has been reduced to a mosaic of patches [9], evidence of the destruction of complex ecosystems. Therefore, protected natural areas (PNA) have emerged to safeguard what remains of natural habitats, aiming to preserve ecological connectivity to mitigate the effects of fragmentation.

In the Peruvian context, the establishment of Protected Natural Areas (PNAs) has not shown optimistic results when faced with the rapid expansion of urban developments, not only in the surrounding areas but reaching a critical point when their physical boundaries are breached, altering the ecosystem’s functionality along with the ecosystem services of the PNA [10,11], as will be presented below. After declaring Peru as the country with the most remarkable diversity of birds on the planet [12], our interest turned toward the conservation status of habitats and resources contributing to the development of bird life cycles on national soil. In the thirteen wetlands located along the Peruvian coastal corridor that are part of the Pacific biological corridor, negative impacts on the wetlands have been documented. These can be categorized as land use conflicts, urban expansion, water availability issues, eutrophication, solid waste accumulation, and flooding [13,14], making the wetlands, according to Ramsar, vulnerable zones that expose bird life to constant danger.

In this context, our study is located in the District of Chorrillos, home to the only coastal wetland in Lima, the Pantanos de Villa Wildlife Refuge (RVSPV) [14]. This wetland hosts 211 bird species (97 resident and 114 migratory) within its various habitats [15,16], out of the 1901 species recorded nationally [17]. Since its designation as Park Zonal No. 25 of Metropolitan Lima in 1987 by D.S. N.009-87 from the Ministry of Housing, its establishment as a PNA (Protected Natural Area) under the Reserved Zone category in 1989, its international designation as Ramsar site in 1997, and its final designation as a Wildlife Refuge in 2006 by D.S. N.055-2006 from the Ministry of Agriculture, integrating it into the National System of Protected Areas (SINANPE) by the Peruvian State, there has been a noticeable process of landscape fragmentation.This process involves the loss and reduction of habitat area (in hectares), and the formation of isolated and disconnected patches [18,19], linked to accelerated urban expansion, as seen in coastal areas [20].

Significant developments contributing to this fragmentation include industrial, commercial, and residential areas, and the construction of local and metropolitan roads that directly border the protected natural area (PNA). Urban expansion has led to increased solid waste pollution and the discharge of effluents into the channels that feed the wetland. Additionally, noise pollution from nearby metropolitan roads poses a further challenge to conserving this ecosystem. Similar scenarios are replicated in various parts of Chorrillos, including areas adjacent and non-adjacent to the RVSPV. Several studies on the RVSPV highlight the need to adapt management strategies to address changes in habitat heterogeneity caused by adjacent urban development [21]. Therefore, it is crucial to investigate the trajectory of loss and fragmentation in the RVSPV, which is immersed in accelerated and chaotic urban expansion. Understanding these changes is critical to safeguarding its biodiversity and reversing landscape degradation. This research is essential not only at the district level of Chorrillos but also at the national level, as it forms part of the Pacific biological corridor.

From this perspective, the study explores the process of natural landscape fragmentation in the Chorrillos district and the RVSPV from 1985 to 2021, focusing on significant changes in urban development during this period and leveraging the availability of data from MapBiomas Peru. This study aims to identify the impact of anthropogenic agents on the study area’s structural connectivity (composition and configuration), driven by accelerated, informal urbanization and incompatible land use that transgresses physical boundaries, particularly in areas adjacent to the RVSPV. While various quantitative analysis methods, including metrics of fragmented patches (shape, size, and connectivity), have been used to estimate alterations in spatial heterogeneity of wetlands [22], this study employs four specific analyses that still need to be conducted in this area. The analysis considers the resilience response of species interacting with the modified spatial heterogeneity in RVSPV [23,24], assesing their survival across spatial and temporal scales during the study period [25].

To conduct this exploration, we employed a combination of two open-access software tools: the MapBiomas Peru Collection 1.0 Platform through Google Earth Engine maps for Land Use and Land Cover (LULC) analysis and the GuidosToolbox (Graphical User Interface for Description of Image Objects and their Shapes–GTB) Version 3.304 for fragmentation analysis. Our approach consisted of the following sections. First, an analysis of Land Cover and Land Use (LULC) to identify the survival of vegetation masses in areas with different land uses. Second, a patch accounting analysis using GuidosToolbox (GTB) was used to assess the location and distribution of patch size classes, allowing for a direct comparison of size distribution across various sites. Third, a Foreground Area Density (FAD) analysis with GTB to determine the extent and types of patches. Fourth, a Morphological Spatial Patterns Analysis (MSPA) using GTB was performed to analyze the shape and connectivity of the identified patches. Through these studies, we aim to provide a quantitative understanding of natural landscape fragmentation due to the loss and fragmentation of wetlands in the Chorrillos district, providing an initial scientific basis for further research on a particular urban wetland.

## 2. Study area

The study area is the Chorrillos district, considered one of the five districts in the Metropolitan Area of Lima that host dense populations of birds. This characteristic of Chorrillos is attributed not only to its location in the coastal region along the Pacific Ocean but also to the presence of a protected natural area (PNA) called the Pantanos de Villa Wildlife Refuge [26]. This wetland ecosystem has been monitored by various researchers for 115 years, recording more than 200 bird species and hosting a wide variety of resident and migratory species, including shorebirds and seabirds that travel thousands of kilometers across different hemispheres following the Pacific migratory route of the Americas [27].

**Fig 1:**
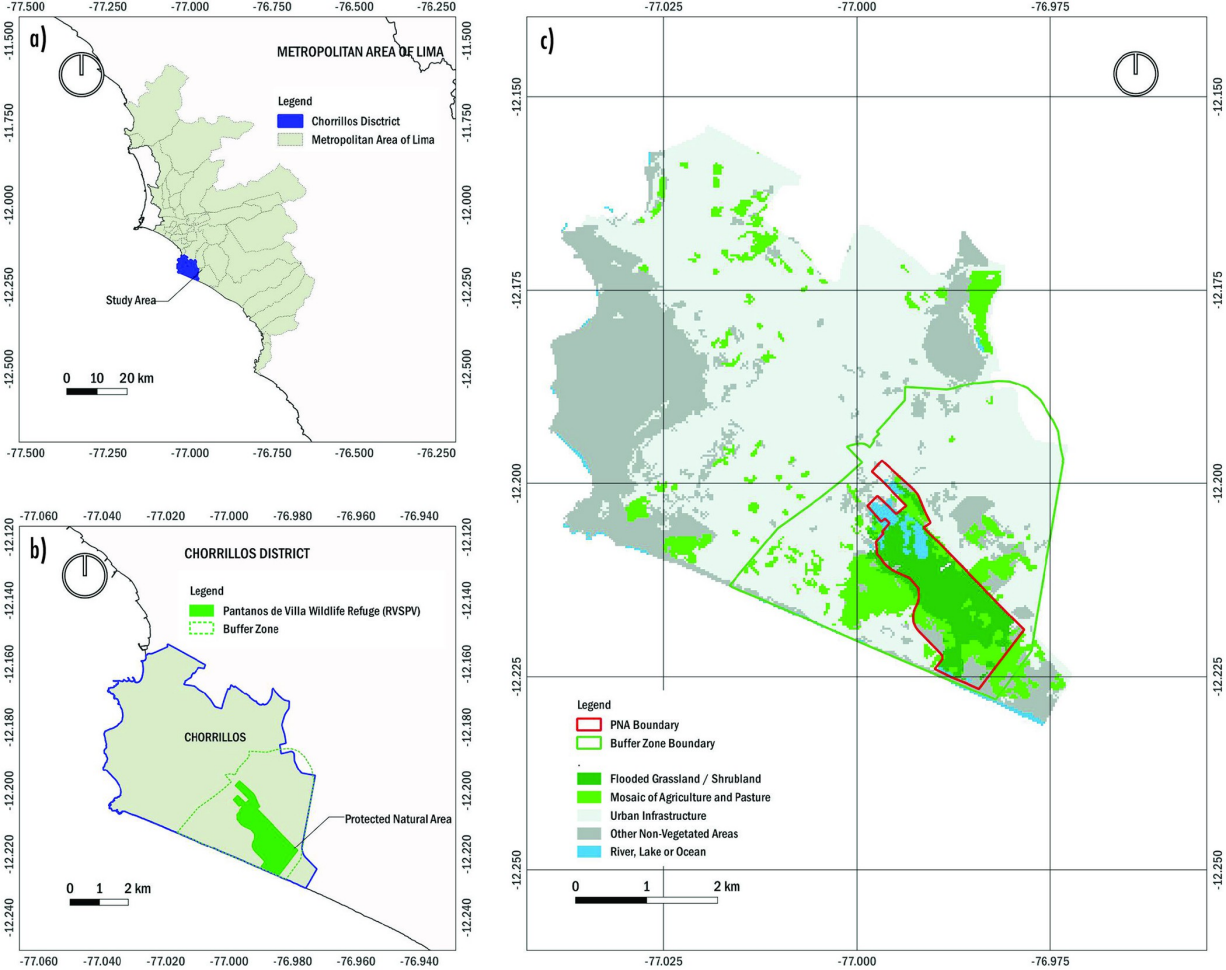
Location of the study area. a) Chorrillos District location in relation to the Metropolitan Area of Lima; b) the RVSPV location and its buffer zone in relation to Chorrillos District; c) Protected Natural Area and buffer zone boundaries in relation to the Flooded Grassland / Shrubland coverage. Fig 1A and 1B were created by the authors using publicly available shapefiles from the geoportal of the National Institute of Statistics and Informatics (INEI) and the geoportal of the National Service of Protected Natural Areas (SERNANP). Data available at https://geoportal.sernanp.gob.pe/ and https://ide.inei.gob.pe/#capas. This drawing has not been previously copyrighted. Fig 1C: Map generated using data from MapBiomas (https://mapbiomas.org) [30].

The Pantanos de Villa Wildlife Refuge (RVSPV) is in the Coastal Desert ecoregion south of Lima. The ecosystem is situated between coordinates 12°10’-12°13’S and 77°01’-77°02’W, with a buffer zone extending over 10.3 km^2^ and surrounded by hills with elevations ranging from 100 to 300 meters above sea level and a linear beach at sea level (Fig 1). Its formation is due to the emergence of groundwater from the old Surco River branch (now channeled) and saltwater infiltration due to its proximity to the coastline. The wetland ecosystem was officially designated as a Wildlife Refuge in 2006 under D.S. N°055-2006-AG, effective January 1 [28], with a protected area of 263.27 hectares [28,29], still considered the largest patch today. Thus, the study area served as a representative sample under the category of Subtropical Pacific Desert Marshes, which hosts important plant communities, migratory and resident avifauna and was recognized as a Ramsar site on January 20, 1997, under the Convention on Wetlands of International Importance Especially as Waterfowl Habitat [13].

The location maps in (Fig 1A and 1B) were constructed using shapefiles obtained from public sources, including the geoportal of the National Institute of Statistics and Informatics (INEI), available at https://ide.inei.gob.pe/#capas, and the geoportal of the National Service of Protected Natural Areas (SERNANP), available at https://geoportal.sernanp.gob.pe/. These data were integrated and edited using QGIS software, ensuring an accurate representation of the study areas. Fig 1C, which shows the distribution of land cover, was generated using data from MapBiomas (https://mapbiomas.org), which is publicly available and authorized for use in scientific research [30].

## 3. Materials and methods

**Fig 2:**
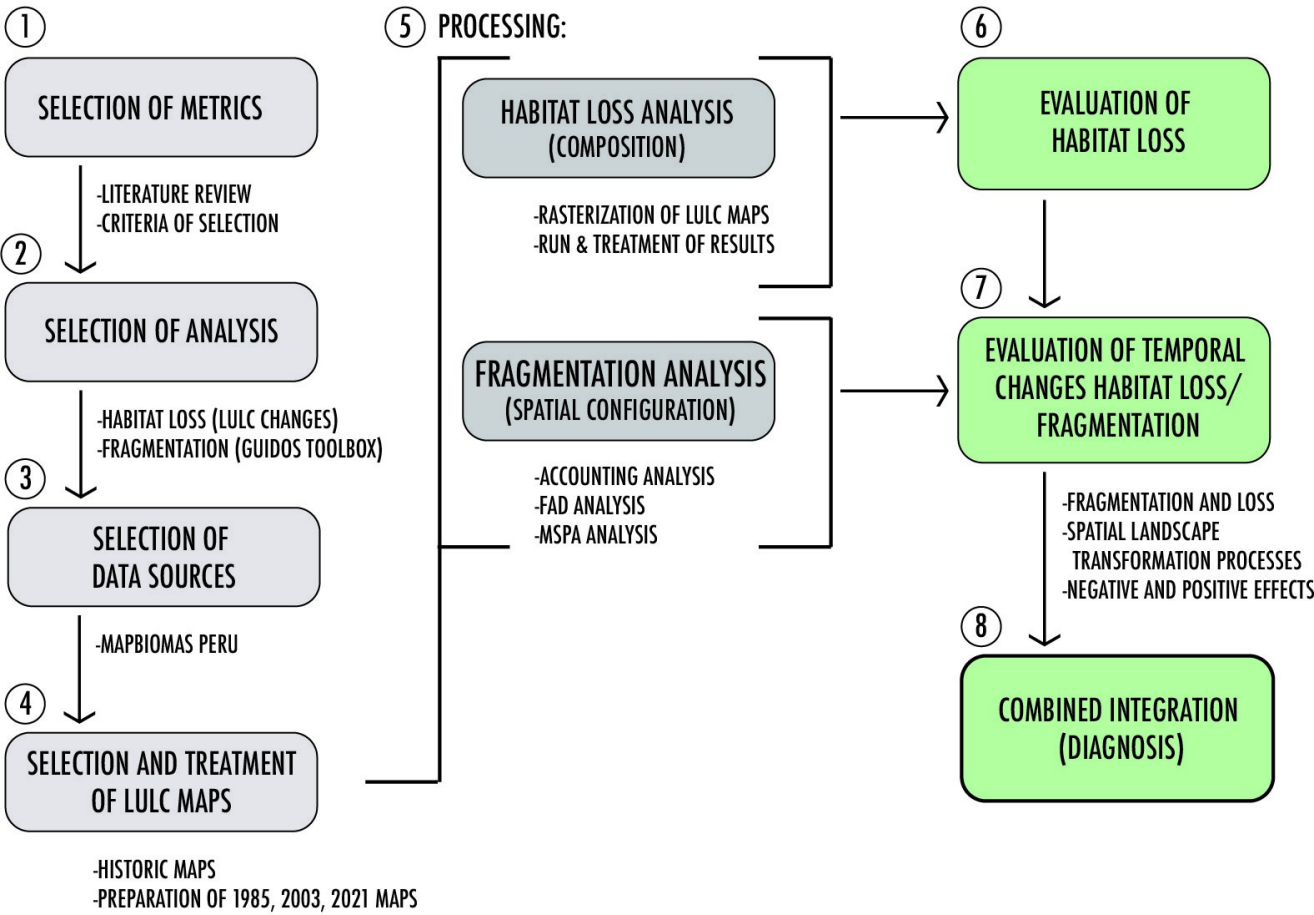
Flowchart of the methodological stages, illustrating the sequence of processes and analyses performed. The authors generated this diagram to outline the study workflow.

Fig 2 outlines the methodological procedures, ranging from choosing metrics and methods to analyze the fragmentation of the natural landscape in Chorrillos to mapping and interpreting the results, based on the study of Babi Almenar, J. et al. [31]. The subsequent subsections provide a detailed description of each stage.

### 3.1 Selection of landscape metrics and fragmentation analysis methods

An initial literature survey was conducted on fragmentation and landscape metrics, including case and comparative studies. Insights from critical articles [32–34] guided the selection of pattern analysis, which employed raster maps incorporating landscape patterns metrics [35]. The objective was to measure and chart different landscape patterns, identify the spatial scales where these patterns occur, and assess their changes over time [36]. Based on the classical conceptual model of "patch-corridor-matrix" [37] and the relevance of wetlands such as the RVSPV in the study area, the focus was on analyzing the fragmentation process through the evaluation of wetland loss and fragmentation. This approach aimed to identify and understand the location, quantity, size, type, connectivity of patches, spatial changes, and the negative and positive effects, allowing them to adapt and coexist in a highly chaotic urban environment.

Our studies used Land Use/Land Cover (LULC) analysis and the GuidosToolbox (GTB) to analyze changes, trends, and the state of fragmentation due to natural or human-induced disturbances [38]. The application of this methodology is promising, given the presence of similar studies analyzing habitat fragmentation in Africa [39] and Asia [40], for example. The difference is that the Landsat image analysis was performed using the LULC data processing of the MapBiomas Peru project maps. This methodology consistently highlights the ecological role of patches and focuses on establishing habitat connections using spatial criteria. It aims to enhance connectivity and ecological interactions between fragments and to counteract landscape loss.

GuidosToolbox (Graphical User Interface for the Description of Image Objects and their Shapes—GTB) is an open-source software for analyzing binary landscapes (habitat versus non-habitat). Created by the European Commission’s Joint Research Centre, Institute for Environment and Sustainability, it facilitates the examination of objects, patterns, and landscape networks. The software features dedicated components for assessing fragmentation and restoration, utilizing various generic raster image processing techniques [41]. Its tools are grounded in geometric principles and are suitable for use across different scales, raster data, and types of natural land cover [41,42]. The compiled versions of the software are available under the GuidosToolbox End User Licence Agreement (EULA) at https://forest.jrc.ec.europa.eu/en/activities/lpa/gtb/, and the open-source code is accessible under GPL version 3 on GitHub (https://github.com/ec-jrc/GTB). This quantitative approach allowed for a detailed analysis of wetland loss and fragmentation.

Spatial changes associated with fragmentation, including decreases in patch size, increased patch isolation, and heightened edge effects, were examined by evaluating the landscape’s composition and configuration across three periods (1985, 2003, and 2021). Using the analysis modules provided by the GTB software, the following aspects were examined: (i) the location and quantity of patches using six size classes from the Accounting module; (ii) the types of patches through 6 classes (intact, interior, dominant, transitional, irregular, and rare) from the Foreground Area Density (FAD) module; and (iii) the connectivity of patches by analyzing seven patterns (Core, Island, Perforation, Edge, Loop, Corridor Bridge, and Background) using the Morphological Spatial Patterns Analysis (MSPA) module. To examine wetland loss, our study utilized the LULC maps for Chorrillos, Lima, provided by MapBiomas Peru. This project offers an annual mapping of land cover and use in Peru, Collection 1.0, from 1985 to 2021.

The Accountability analysis provides the location and distribution of six classes of foreground patch sizes on a given map. These classes are defined based on the number of pixels, starting with Class 1 for the smallest object (1 pixel) and extending to Class 6 for the largest objects. The module automatically adjusts each foreground patch into its respective size class [41]. Foreground patches are color-coded according to the defined categories. The Foreground Area Density (FAD) analysis evaluates fragmentation by assessing the density of foreground areas surrounding pixels [43] across five defined observation scales (rare, patchy, transitional, dominant, interior, and intact). For this study, the definition of "foreground" considered the coverage and land uses of the Agricultural Mosaic and the Flooded Grassland / Shrubland to determine fragmentation. The FAD analysis measures the pixel density in the foreground, assigning a low-density value to edge pixels to identify fragmented areas versus preserved ones. The six basic fragmentation classes FAD provides are summarized in Table 1 [42,44]:

**Table 1:** Fragmentation classes (FAD-APP 6-class) and definitions provided by the GuidosToolbox (GTB).

FAD-APP 6-Class	Range	Definitions
**Rare**	FAD < 10%	Rare patches of fragmented natural land cover class.
**Patchy**	10% ≤ FAD < 40%	Dispersed patches of natural land cover class without clustering.
**Transitional**	40% ≤ FAD < 60%	Patches of natural land cover class experiencing habitat changes.
**Dominant**	60% ≤ FAD < 90%	Patches of natural land cover class frequently found in fragmented habitats.
**Interior**	90% ≤ FAD < 100%	Areas featuring a core of a specific size display central characteristics.
**Intact**	FAD = 100%	Natural land cover class without changes.

Using mathematical morphological techniques, the Morphological Spatial Patterns Analysis (MSPA) classifies and describes landscape spatial patterns with notable computational efficiency [30,42]. This method categorizes habitat areas into seven types: core, islet, perforation, edge, loop, bridge, and branch, with particular emphasis on structural connectors (bridges), which are elements without interior area (for the specified edge distance) that connect at both ends to habitat patches that do have such interior area [45]. Table 2 [42] provides a summary of the seven fundamental morphological pattern classes identified by MSPA:

**Table 2:** Foreground classes for MSPA and morphologic definitions by GTB; and landscape ecological definitions by Liang, Z.; & Feng, X. [40].

MSPA Classes	Morphologic Definitions	Landscape Ecological Definitions
**Core**	Interior area excluding the perimeter.	Extensive habitat patches that function as source areas, offering habitats or migration routes for wildlife.
**Islet**	Disjoint and too small to have a core.	Small, loosely connected patches that facilitate species dispersion and interaction and enhance the flow of matter and energy.
**Perforation**	Perimeter of the interior area.	The transitional zone between the core area and the non-vegetated landscape is characterized by the edge effects of the inner patch.
**Edge**	Perimeter of the outer area.	The buffer zone between the core area and the non-vegetated landscape exhibits border effects and safeguards the ecological functions of the core area.
**Bridge**	Connected to different central areas.	Corridors linking adjacent core area serve as routes for species movement and energy transfer between neighboring core patches.
**Loop**	Connected to the same central area.	Links corridors within a single core area, facilitating species movement and energy flow within the core patch.
**Branch**	Perforation.	Connected on only one side to an edge, bridge, loop, or perforation.

### 3.2 Data selection and processing of land use/land cover maps

Land use/land cover class maps were sourced from the MapBiomas Peru project, an online database offering free Land/Use/Land Cover (LULC) data to assess structural connectivity in the landscape. The LULC data were selected from the MapBiomas Peru project due to its availability as a continuous, high-resolution spatial dataset covering the period from 1985 to 2021. This dataset accurately represents land cover changes in the study area, crucial for long-term fragmentation analysis. Additionally, MapBiomas data have been validated by experts, ensuring their accuracy and reliability for scientific research. Furthermore, the formats provided by MapBiomas are compatible with GuidosToolbox (GTB), facilitating seamless integration into the analyses, such as Accounting, FAD, and MSPA, which enhances the reproducibility of the methods employed.

This dataset, which spans from 1985 to 2021, is derived from annual Landsat images with a 30-meter spatial resolution, presented as mosaics covering the entire country of Peru, and is part of Collection 1.0. The LULC maps were created using a Random Forest classifier on the Google Earth Engine platform [46]. Due to its heterogeneity, this classification method employs a machine-learning algorithm and reports high accuracy, even in complex scenarios. Using the Random Forest algorithm as a classifier for the LULC maps in Collection 1.0, combined with a flexible mapping protocol, allows each country in various initiatives to define its feature space and samples. Post-classification filters were also applied to reduce effects associated with low-quality and limited availability of satellite images, primarily occurring at the beginning of the time series [47]. The datasets analyzed for LULC maps in this study are publicly available through MapBiomas Perú, accessed on December 28, 2023. (https://plataforma.peru.mapbiomas.org) [46]. These datasets can also be accessed directly on Google Earth Engine using the ID: *projects/mapbiomas-public/assets/peru/collection2/mapbiomas_peru_collection2_integration_v1*. Additionally, the processing scripts used by MapBiomas Perú are available on GitHub (https://github.com/mapbiomas-peru). This study applied a quantitative approach to analyze the land use and cover changes based on these datasets.

Utilizing the LULC maps from the MapBiomas Peru Collection 1.0 available on Google Earth Engine enabled their export according to the political level 4 boundaries (municipal divisions) for analysis specific to the Chorrillos district. The maps selected for analyzing the fragmentation process in the natural landscape of Chorrillos and the RVSPV correspond to the years 1985 (the year LULC maps began), 2003 (intermediate year), and 2021 (the final year of the Collection 1.0 LULC maps). The LULC classification in Collection 1.0 MapBiomas Peru used sixteen land use classes considering two levels of class and five types of biomes, with an additional class for unobserved areas that could not be classified due to cloud presence, cloud shadow, atmospheric noise, or image quality [47]. Situated in the Coastal Desert biome, the study area used a classification of six land cover and land use types: dry forest, flooded grassland/shrubland, mosaic of agriculture and pasture, urban infrastructure, other non-vegetated areas, and river, lake, or ocean. Table 3 [47] summarizes these six fundamental land cover and land use categories from MapBiomas Peru.

**Table 3:** Landscape coding types and definitions of LULC classes by MapBiomas Peru.

Coding	Types	Definitions
**4**	Dry Forest	Forested cover is located in areas adjacent to watercourses.
**11**	Flooded Grassland / Shrubland	Vegetative cover along the coastal shoreline. It is characterized by herbaceous vegetafromo periodic flooding, including swampy areas temporarily flooded by El Niño events.
**21**	Mosaic of Agriculture and Pasture	Areas used for agriculture and livestock, separating agriculture and pasture classes was impossible. It includes green areas within urban zones.
**24**	Urban Infrastructure	Areas covered by urban infrastructure and all associated green spaces and communication routes make up an urban system.
**25**	Other Non-Vegetated Areas	Areas with little or no natural vegetation, such as sandy soils or rocky outcrops. It also includes anthropogenic areas such as urban and roadway infrastructure; soils exposed by logging or mining and natural landslides; burned areas; and non-photosynthetic herbaceous covers of natural or cultivated grasses.
**33**	River, Lake, or Ocean	Any expanse of water on the Earth’s surface, whether natural or artificial. It includes rivers, lakes, reservoirs, ponds, and other bodies of water.

Moreover, the current study focuses on quantitative spatial analysis of wetland fragmentation and does not incorporate stakeholder engagement as part of its methodology. While dialogues with local stakeholders were conducted, we plan to use this qualitative data in a future publication to explore the socio-economic factors more deeply. The plan is to provide a more comprehensive understanding of the human dimensions related to wetland degradation in a separate, focused analysis.

### 3.3 Data processing for wetland loss and fragmentation analysis

For calculating wetland loss (landscape composition), the analysis was based on LULC maps obtained from the MapBiomas Perú platform, Collection 1.0 [46]. The maps were converted to vector format using QGIS v3.03, and the metrics values for each patch were integrated with their corresponding attribute tables. To calculate wetland fragmentation (spatial configuration of the landscape) through an understanding of patches—defined as areas of variable size with different physiognomy compared to their surroundings and a degree of internal homogeneity [47]—the methods used were Accounting, FAD, and MSPA from the GuidosToolBox (GTB). For this, the input map must be BYTE type and contain two data classes: Foreground and Background. In classifying the fragmentation process on a wetland map, the Foreground represents wetlands, and the Background represents non-wetlands. Generally, the Foreground corresponds to the features that need to be classified, while the Background complements the Foreground.

Additionally, there may be data that are neither Foreground nor Background or should be excluded; these belong to the Missing class. The process begins by binarizing the existing land use raster data, assigning a value of 1 byte to Background (non-wetland) and 2 bytes to Foreground (wetland). This study considers Flooded Grassland / Shrubland and Agricultural Mosaics as Foreground categories for analyzing wetland loss and fragmentation.

The current analysis period (1985–2021) was determined based on the availability of high-quality, validated data from the MapBiomas Peru project, which offers continuous LULC data over this period. This period allows us to conduct a robust analysis of wetland fragmentation and changes in land use. While a more extended longitudinal analysis could provide additional insights, the available data from MapBiomas covers the most relevant temporal scope for understanding long-term changes in the study area. We acknowledge that future research could explore a more prolonged timeframe if more historical or future data becomes available. Although we obtained permits for field site access from the National Service of Protected Natural Areas (SERNANP) and the Municipal Authority of the Pantanos de Villa (PROHVILLA), the fieldwork conducted under these permits is not included in the methodology of this paper. This fieldwork will be part of a future study. The current study relies exclusively on spatial analysis using remote sensing and land cover data, and no direct field interventions were performed in this work.

## 4. Results

### 4.1 Wetland loss from 1985–2021

**Fig 3:**
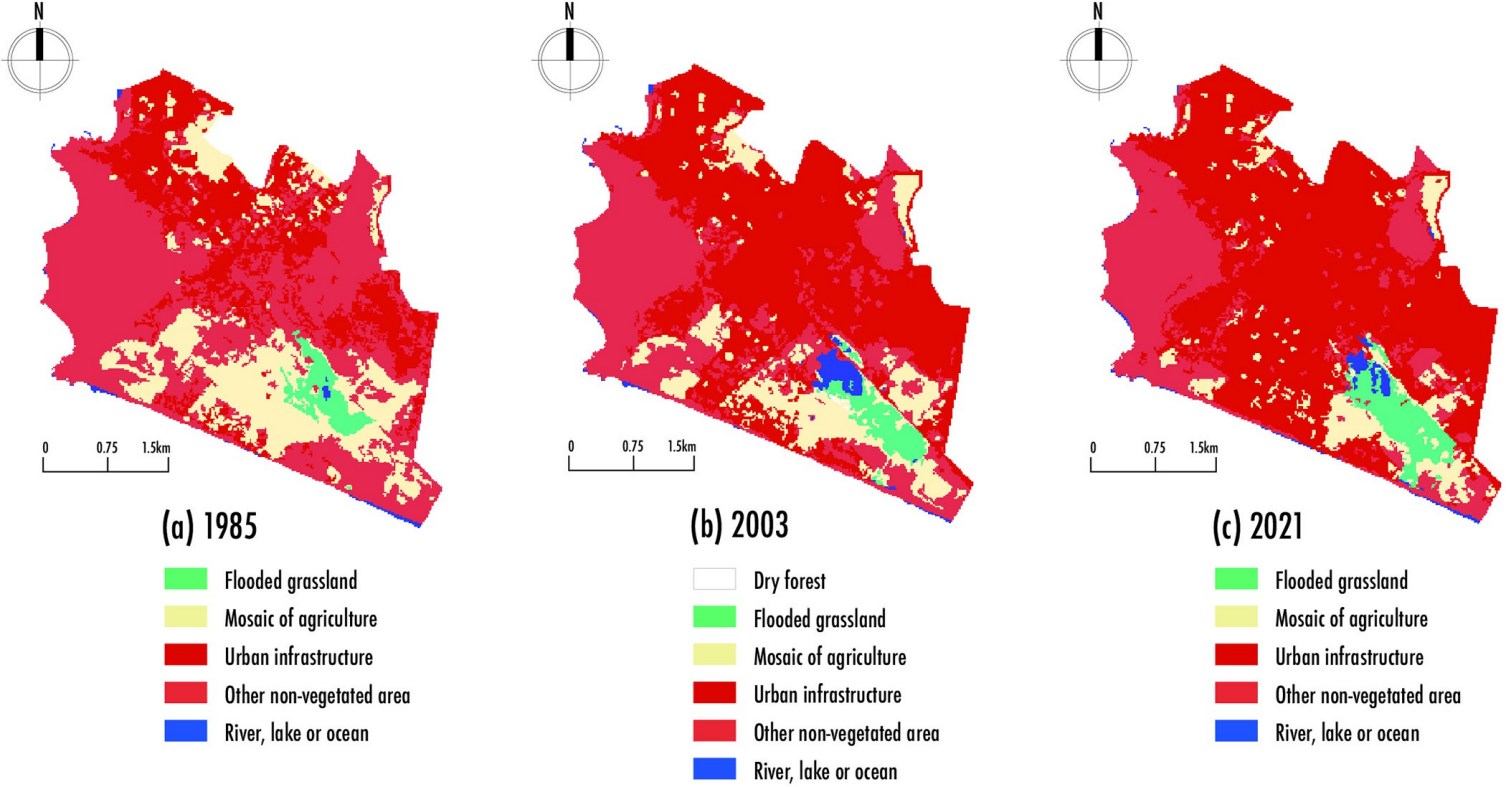
Land Use and Land Cover (LULC) 1985–2021 using MapBiomas Peru. a) LULC 1985; b) LULC 2003; and c) LULC 2021, generated with data from MapBiomas (https://mapbiomas.org) [30]. Data were processed using publicly available datasets from MapBiomas, which were open for scientific research.

The loss of landscape in Chorrillos district and the RVSPV was visualized through the coverage and land use of the Agricultural Mosaic and the Flooded Grassland / Shrubland area, which represents the wetland vegetation during the study period from 1985 to 2021 and is mainly located in the southern part of Chorrillos. Fig 3. shows the distribution of land cover, which was generated using data from MapBiomas (https://mapbiomas.org) [30], publicly available and authorized for use in scientific research. Over 37 years, a gradual upward trend was observed in the loss of Agricultural Mosaic coverage. At the same time, there was a gradual upward trend in the increase of coverage termed Flooded Grassland / Shrubland area, as summarized in Table 4. In the first period (1985–2003), Agricultural Mosaic coverage decreased by 249.62 hectares, whereas in the second period (2003–2021), the loss was 193.05 hectares. Fig 3A–3C illustrate the Agricultural Mosaic coverage in the northern region and the coastal edge of the southern part of the district. Conversely, the Flooded Grassland / Shrubland area increased by 4.37 hectares during the first period and 55.69 hectares during the second period. The location of the Flooded Grassland / Shrubland area coverage is in the southern part of the district, where it grew until it bordered a section of the coastal edge.

**Table 4:** Land use and land cover change analysis. Source: GuidosToolbox (GTB).

Land Use/Land Cover	Area in 1985 (ha)	Area in 2003 (ha)	Area in 2021 (ha)	% Change in LULC (1985–2021)
**Dry Forest**	0	3.32	0	0
**Flooded Grassland / Shrubland**	102.90	107.27	162.96	+ 1.62
**Mosaic of Agriculture and Pasture**	769.04	519.42	326.38	- 11.92
**Urban Infrastructure**	938.81	1798.71	2250.12	+ 35.31
**Other Non-vegetated Areas**	1879.98	1219.42	922.86	- 25.77
**River, Lake, or Ocean**	17.75	59.45	46.07	+ 0.76

Although the total estimated area of loss of Agricultural Mosaic coverage was 442.66 hectares (11.92%), the total estimated area of increase in Flooded Grassland / Shrubland coverage observed was 60.06 hectares (1.62%) during the study interval (1985–2021) as summarized in Table 4. The result of the loss of Agricultural Mosaic was concentrated in the southern part of Chorrillos district. At the same time, an increase in Flooded Grassland / Shrubland coverage was recorded, mainly consisting of the RVSPV and the Chira relic. This increase left the remaining plant cover of the district primarily as wetlands in the south.

### 4.2 Wetland fragmentation 1985–2021

The analysis was conducted using GuidosToolbox (GTB) for Accounting [41], FAD [41], and MSPA [42,48], as detailed in the GTB documentation.

#### 4.2.1 Accounting analysis

**Fig 4:**
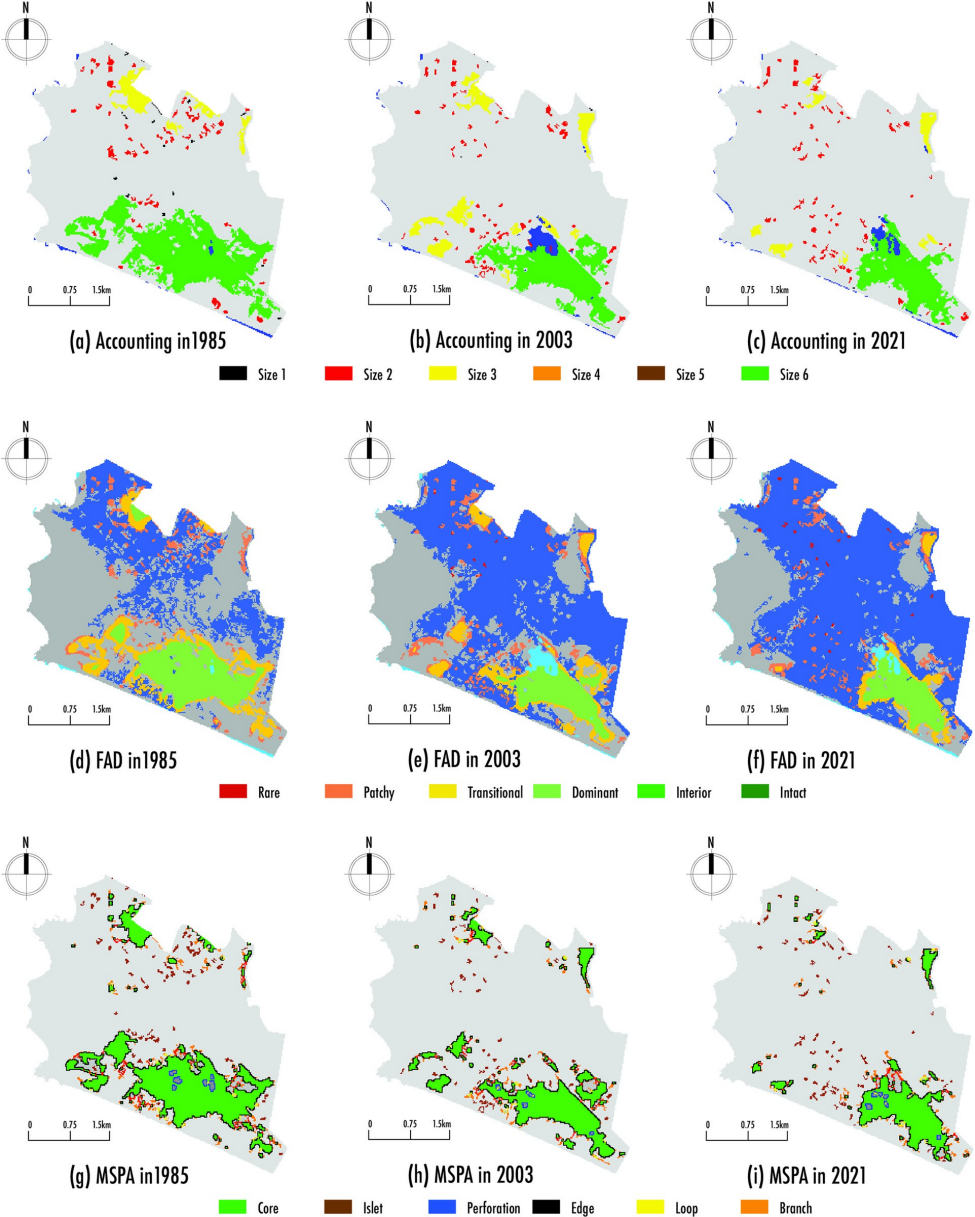
Wetland fragmentation 1985–2021 using GuidosToolbox. a) Accounting Analysis [41]; b) Foreground Area Density (FAD) Analysis [41]; c) Morphological Spatial Patterns Analysis (MSPA) [42,48]. Data were processed with GuidosToolbox, a free and open-source software. Available at: https://forest.jrc.ec.europa.eu/en/activities/lpa/gtb/.

The quantity and size of patches varied during the studied period of 1985–2021. Fig 4A–4C display four patch sizes: size 1, size 2, size 3, and size 6, while size 4 and size 5 showed no observable change, maintaining 0 objects throughout the study interval (1985–2021). Size 1 is the smallest, and size 6 is the largest, allowing for identifying fragmentation hotspots. This classification enables us to designate size six as major patches, size three as intermediate patches, and sizes 1 and 2 as minor patches.

In 1985, 75 patches were recorded with a combined area of 10,046 hectares. Among these, one patch was categorized as a major patch with an area of 7,902 hectares, four were intermediate patches totaling 1,147 hectares, and the remaining 70 were minor patches with an area of 997 hectares, as shown in Fig 4A. Compared to the patches in 2003, the total number remained the same at 75 patches. However, the total and major patch areas decreased to 7,230 hectares and 4,392 hectares, respectively. On the other hand, the intermediate patches increased to 10 patches with a total area of 1,990 hectares, while minor patches showed a slight decrease, totaling 64 patches and 848 hectares as seen in Fig 4B. In 2021, despite the rise in the number of patches to 78, the total area and the area of the largest patch decreased, measuring 5,610 hectares and 3,800 hectares, respectively. Similarly, Fig 4C shows a decrease in intermediate patches, now totaling seven patches with an area of 923 hectares. In contrast, minor patches increased, with 70 patches similar to those in 1985 but with smaller sizes, totaling 4 hectares.

**Fig 5:**
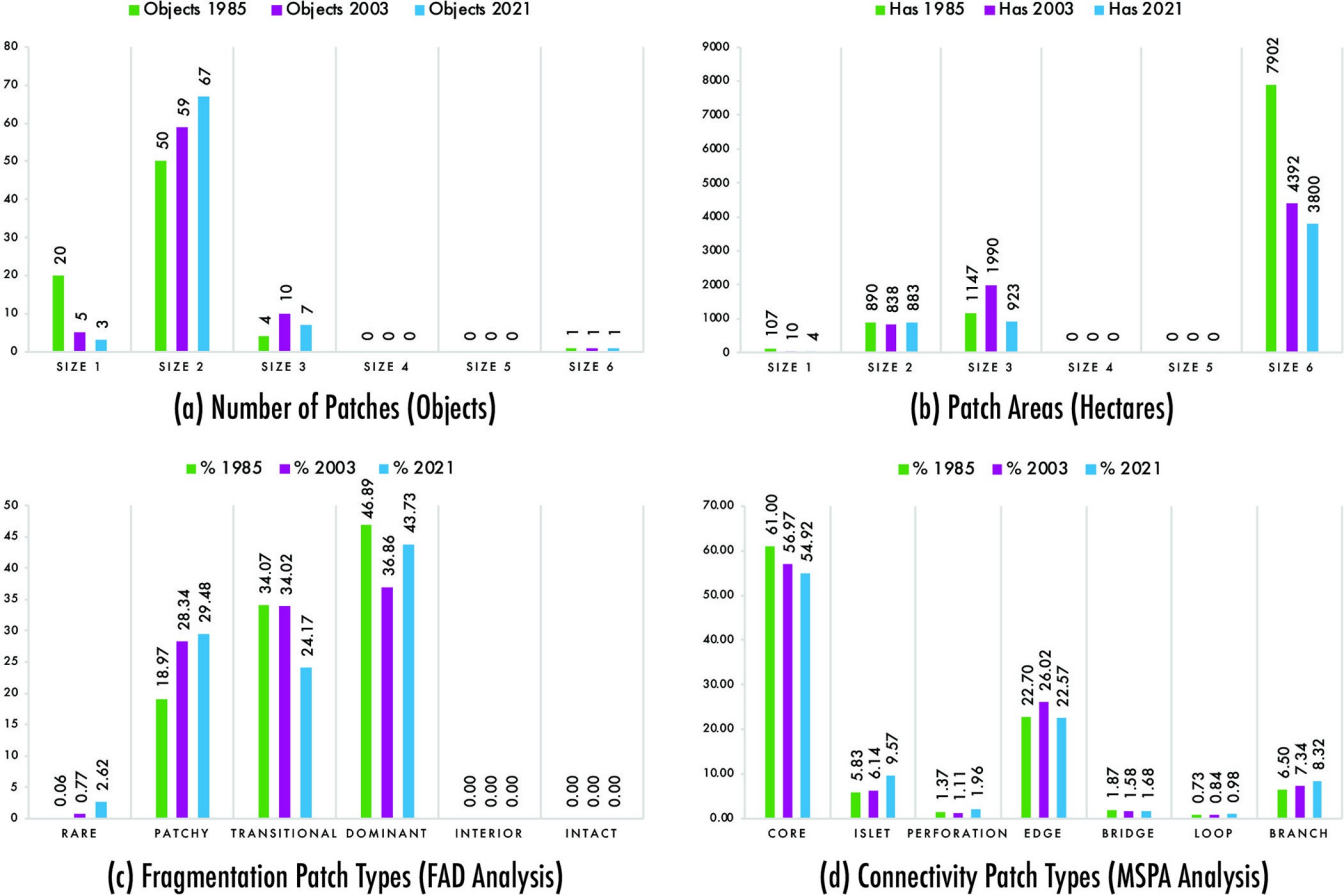
Wetland fragmentation change analysis using GuidosToolbox. a) Number of Patches (Objetcs); b) Patch Areas (Hectares); c) Fragmentation Patch Types (FAD Analysis); d) Connectivity Patch Types (MSPA Analysis). Data were processed using GuidosToolbox, which provides tools for landscape pattern analysis. Available at: https://forest.jrc.ec.europa.eu/en/activities/lpa/gtb/.

During the study period (1985–2021), there is clear evidence of a loss in the area of patches across all recorded sizes. Although the major patch has remained the largest compared to the others, its area has decreased by 48%, concentrating on the RVSPV. Fig 5A and 5B show that intermediate and minor patches are created as the size of the major patch decreases. Regarding intermediate patches, 2003 saw the highest number and area, representing 54% of the total area and mainly located on the side adjacent to the sea. For minor patches, both in 1985 and 2021, the number of objects remained the same, situated on the side adjacent to the sea and in the northern part of the Chorrillos district, but with different areas and a loss of 110 hectares.

#### 4.2.2 Foreground Area Density (FAD) analysis

The classes of fragmentation in the patches changed over the years examined. Fig 4D–4F show four types of fragmentation classes throughout the Chorrillos district: dominant, transitional, patch, and rare between 1985 and 2021, while the inferior and intact classes showed no observable change, maintaining 0% throughout the study interval (1985–2021).

The FAD analysis for 1985 reveals that the study area had 47% dominant cover and 34% transitional cover, both located in the northern part of the district but especially prevalent in the southern part of the district (RVSPV), with 19% of patch cover distributed sparsely throughout the study area, as shown in Fig 4D. For 2003, there was 37% dominant cover, 34% transitional cover, and 28% patch cover, maintaining the primary concentration in the southern part of the district (RVSPV), with 1% rare cover located in the northern part as seen in Fig 4E. Compared to 1985, the decrease of 10.03% in the dominant cover within the wetland led to a slight decline of 0.05% in the transitional cover, a 9.37% increase in patch cover, and a slight increase of 0.71% in rare cover. In 2021, Chorrillos district has 44% dominant cover, primarily located in the southern part of the district (RVSPV), 29% patch cover distributed sparsely throughout the study area, 24% transitional cover in the northern area (private military park functioning as a cemetery) and southern area (RVSPV and the Chira remnant), and 3% rare cover, most of which is located in the northern part, as shown in Fig 4F. Relative to 2003, there was a 6.87% rise in dominant cover extending towards the beach, a 9.85% reduction in transitional cover, a 1.14% increase in patch cover, and a 1.85% increase in rare cover.

The increases observed during the study intervals (1985–2021) were in the rare and patch cover classes, which contain patches in fragmented wetlands and dispersed patches without clustering, with increases of 2.56% and 10.51%, respectively, as shown in Fig 5C. On the other hand, decreases were noted in transitional and dominant cover classes, where patches experience habitat changes and are more frequently found in fragmented habitats, with decreases of 9.90% and 3.17%, respectively.

#### 4.2.3 Morphological Spatial Patterns Analysis (MSPA)

Fig 4G–4I display the distribution of MSPA patterns in the study area from 1985 to 2021, highlighting seven spatial structure metrics: core, islet, perforation, edge, loop, bridge, and branch [42,48].

In 1985, the core layer had the highest representation at 61.00%, located primarily along the coastal edge of the southern part of the district, making it the record with the highest concentration of large habitat patches, as shown in Fig 4G. Compared to other years, 2003 and 2021 showed a continuous decrease in the core layer, with 56.97% and 54.92%, respectively. The loss of the core layer during the study period (1985–2021) was 6.08%, with the highest concentration in the southern area (RVSPV), as shown in Fig 5D. Consequently, the islet layer progressively increased due to a decreased core layer, with a dispersed growth pattern. In 2021, a 9.57% increase was recorded, the highest value, with a distribution of very dispersed islet patches, most located in the northern and southern areas as seen in Fig 4I. In comparison, 1985 and 2003 recorded 5.83% and 6.14%, respectively, maintaining the highest concentration of islet patches in the district’s north and southern areas (RVSPV). The growth of the islet layer during the study period (1985–2021) was 3.74%, as shown in Fig 5D.

In 2021, the highest proportion of perforations was observed at 1.96%, marked by numerous perforation areas disconnected from water bodies within the RVSPV. This indicates a significant prevalence of perforations in the large wetland patches, as shown in Fig 4I. The growth of the perforation layer during the study period (1985–2021) was 0.59%. In terms of edges, 2003 exhibited the largest edge area at 26.02%, whereas 1985 and 2021 had 22.70% and 22.57%, respectively. This trend indicates more intricate green areas’ edge shapes and heightened exchange of matter and energy. The growth in the loss of the perforation layer during the study period (1985–2021) was 0.13%, as shown in Fig 5D. Throughout the study period (1985–2021), the proportion of bridge and loop areas in Chorrillos remained low, reflecting a lack of overall landscape connectivity for wetlands in the district. However, in 2021, the highest proportion of loop area was observed at 0.98% and bridge area at 1.68%.

During the study period (1985–2021), the loop layer increased by 0.25%, aiding species movement within patches. Despite a 0.19% reduction in the bridge layer, the Chorrillos district maintains robust landscape connectivity, especially in the southern region of RVSPV. The area covered by corridor branches increased from 6.50% in 1985 to 8.32% in 2021, representing a growth of 1.82%. Meanwhile, Fig 4G–4I reveal a reduction in the core layer. This decline is associated with an increase in the perforation layer, which delineates the edges of internal areas between fragments. Additionally, decreases in the edge and bridge layers point to a reduction in wetlands around the outer perimeter of the ecosystem.

## 5. Discussion

The study is the first to explore the natural landscape fragmentation in the Chorrillos district and the RVSPV from 1985 to 2021, focusing on how anthropogenic agents affect the area’s structural connectivity, including composition and configuration. We achieved this by combining LULC analysis with the GuidosToolbox (GTB). While diverse methods and metrics are available for this field study, our analysis offers a novel perspective by addressing three key aspects: i) shifts in fragmentation and loss, ii) links to spatial landscape transformation processes, and iii) the effects of fragmentation.

### 5.1 Shifts in fragmentation and loss

Based on the analysis of LULC maps, Accounting, FAD, and MSPA for the Chorrillos district and the significant patch of the RVSPV from 1985 to 2021, we found that the habitat fragmentation process confirms an ongoing dynamic process. This process is marked by four key elements established in the literature: a) extensive habitat loss across the landscape; b) a decrease in the size of the habitats that remain; c) greater isolation of habitats due to new land uses encroaching on intervening areas [49,50]; and d) a rise in the quantity of smaller patches. Habitat fragmentation has reduced and divided habitats into smaller, isolated patches, affecting species’ dynamics [51]. Changes in vegetation cover have negatively impacted species survival and the adaptability of organisms due to their specific responses to ecological [39] and anthropogenic pressures, affecting local biodiversity [52]. This pattern of habitat loss in coastal wetlands is not unique to Lima but is common in other densenly populated coastal cities undergoing urbanization [20].

While the RVSPV has remained the largest patch (classified as size 6), it has experienced a continuous decrease in its area, formed by the coverage and land uses of the agricultural mosaic, which has been identified as one of the most common causes in the fragmentation process [52]. In contrast, the coverage of the Flooded Grassland / Shrubland patch has slightly increased. This patch is initially located along the coastal edge and now primarily encompasses the RVSPV wetland area and the private club adjacent to the beach. The fragmented Dominant patch class, with Transition class edges, further evidences the fragmentation process, as these classes are associated with fragmented habitats experiencing habitat changes. Consequently, the large patch, considered the Core layer, has shown a decline in its extensive natural reserves, potentially limiting habitat and migration opportunities for wildlife.

Regarding the Edges layer of the large patch, its constant land use changes due to zoning adjustments and incompatible land use [3,53] make it more susceptible to anthropogenic disturbances. One possible explanation is its proximity to industrial belts, sports club installations, and other infrastructure that have progressively encroached on the ecosystem, demonstrating the complexity of green space contours [40], which limits the protective capacity of the ecological process of the natural reserve and produces edge effects. Additionally, a small number of Perforation layers within the large patch increases non-habitat uses within large patches [40,54]. Another possible explanation is the presence of properties with their respective constructions [53] that were in place before the area became a PNA, contributing to the generation of edge effects.

The decrease in the large patch area and the alteration of ecosystem function dynamics resulted in fragmented landscape patterns, with an increase in isolated intermediate and smaller patches that separate and reduce habitat quality. This rise in the number of intermediate and smaller patches in the study area has been disproportionate to the overall area, as it recorded an increase in area loss primarily from the Agricultural Mosaic land cover and land uses. Fragmented patch classes such as Patches and Rare show increases and dispersion due to rapid urban expansion (both planned and unplanned) and land use changes. In terms of connectivity, the number of Islet-class patches has increased. Even though patches have weaker connections, they still facilitate the movement of matter and energy. Despite a decline in Bridge-class patches, which link various patches [40], the rise in Loop and Branches-class patches, especially those connecting to the large patch, has enhanced species dispersal and energy flow. This indicates a strong connectivity.

The loss of habitat within the fragmented landscape disrupts its quality, affecting the Core and Bridge patches, which are crucial for wildlife dispersal and species migration and vital for ecosystem health and natural regeneration [14]. This is not uncommon in developing countries, where uncontrolled urban growth affects natural ecosystems, as seen in the wetlands of Concepción, Chile, for example [55]. Our findings are consistent with previous studies, which showt a decline in wetland integrity over time, and demonstrate how human activities have significantly altered the study area, negatively impacting the ecosystem’s capacity to provide shelter, resting, feeding, and breeding habitats for bird species.

### 5.2 Links to spatial landscape transformation processes

As a result of these landscape change patterns during the study period (1985–2021), our findings identified six spatial changes from the ten spatial processes for landscape transformation [6] defined by Bogaert, J. et al. [6]: attrition, creation, dissection, fragmentation, perforation, and shrinkage related to the process of fragmentation. Using the Decision Tree Algorithm to Identify Spatial Processes by Bogaert J. et al. [6], we analyzed these spatial processes in Chorrillos by examining changes in three variables: area or size, boundary length, and quantity of patches. For a more precise understanding, these were reviewed from a general perspective, focusing on the large patch and a perspective on smaller patches, across two intervals 1985–2003 and 2003–2021.

From a general perspective, three spatial patterns emerged: Perforation in the first interval and Dissection and Fragmentation in the second interval. The typical loss of area characterizes these patterns. The Perforation pattern is marked by a decrease in area and an increase in perimeter. One explanation for this is the presence of gaps in the main patch with continuous growth. In the second interval, a Dissection pattern emerged, characterized by an increase in the number of patches and a reduction in area due to the highway (metropolitan avenue) cutting through and splitting the main patch into two. Despite a significant habitat loss of 78%, the resulting numerous separate patches of varying sizes and shapes confirm the landscape transformation, leading to increased fragmentation of the land cover.

From the perspective of the large patch, two spatial patterns were identified: Perforation in the first interval and Shrinkage in the second interval. Under this approach, the initial Perforation pattern exacerbated the increase in the edge effect within the large patch as it encountered non-habitat areas created within it. However, the subsequent Shrinkage pattern observed reveals that despite the main patch’s status as a single patch, it experienced a progressive loss of area and perimeter. Consequently, one possible explanation is that the large patch weakly maintains its original shape with no apparent "wear and tear."

Two spatial patterns were observed for the smaller patches: Attrition in the initial interval and Creation in the subsequent one. Due to their disappearance, the smaller patches reflect the final habitat loss and fragmentation. stage Conversely, the Creation pattern involved the emergence of new smaller patches and an expansion of their area. This range of spatial transformation processes suggests a fragmented distribution of large wetland patches and a more scattered arrangement of smaller wetland patches. It highlights the impacts of fragmentation and loss, which are further detailed below, focusing on the large patch.

### 5.3 Effects of fragmentation

In light of the conversion of wetland areas to urban infrastructure uses, it is pertinent to question the impact of this rapid urbanization on wetlands. Previous research has shown that the loss of agricultural lands due to the decline in the water table over 25 years until 1999 [53] led to the disappearance of the natural filtration processes in farmland irrigation [56]. This loss facilitates the conversion of arid lands for urban development. Consequently, the accelerated growth of this area was driven by the construction of informal settlements and beachfront condominiums or planned settlements built on cleared wetlands and surrounding areas [1,53], which have significantly altered the natural conditions of the ecosystem [57].

Urban development also necesitated city infrastructure development, including transportation networks. One possible explanation for the isolation of the large patch in the Chorrillos district is the construction of one metropolitan road (Avenida Huaylas) and one local road (Avenida Alameda Las Garzas Reales). The characteristics of these roads, including their proximity to the protected natural area, have resulted in the complete isolation of the patch. This isolation continues to affect the wetland due to the loss of land cover [58] and the movement of species, possibly reducing bird populations (due to food scarcity and lack of resting areas) and their dispersal, as their edges are left unprotected and they lose ecological connectivity.

Focusing on the edges of patches and identifying their potential causes of fragmentation due to various land uses is essential. Studies on Lima’s coastal wetland ecosystems have identified several driving patterns such as land use change [58], accumulation of debris or garbage, degradation due to livestock and grazing, and species introduction [59], mainly occurring in the edge layer. Due to its ecological connectivity value, this study focuses on the edge effects of the major patch (RVSPV), particularly where its perimeter abruptly borders areas with incompatible land uses, such as transportation infrastructure that dissects the major patch into two parts. Another possible explanation is that the layout of metropolitan and local avenues becomes disturbing boundaries for the major patch, causing possible reductions in the expansion area and disturbances towards its interior without escaping from the different types of pollution they generate. Noise pollution from vehicular traffic creates zones that are avoided by bird species sensitive to sound, as noise can disrupt their vocal communication [60,61]. Light pollution (whether mobile or fixed) affects birds by altering their activity and behavior patterns, as seen with migratory species [62]. Public transportation infrastructure generates access flows that can threaten the PNA if users are not adequately trained. Consequently, the effects concentrated on the margins of the large patch create a perimeter-to-area ratio [63], potentially generating more low-quality bird species habitats (at the edge).

Despite the negative impacts of fragmentation in the study area, our findings reveal an optimistic aspect: the persistence of water links in the major patch. This patch contains the only lagoon that serves as a resting area for migratory birds. Although this lagoon faces contamination from solid waste and untreated sewage [64,65], it has continued to support migratory birds by providing essential feeding grounds through underground water channels [56], which previous researchers focused on. In other words, the persistence of this water body has maintained structural connectivity, linking different wetland patches, while adapting to changes in spatial configuration (land cover types) and contamination. These natural links/corridors facilitate the flow of life in the form of materials and organisms from one place to another [66]. Mitigating the effects of isolation enables the development of the ecological connectivity of the largest patch, which is assigned the role of a “stepping stone”. Despite fragmentation, the major patch remains a vital refuge for resident and migratory bird species. This type of structural recognition motivates further research and promotion of restorative functions that benefit connectivity while avoiding and/or minimizing barriers that disrupt the functionality of natural refuges [67].

## 6. Conclusions

From an ecological perspective, understanding the functioning of a landscape requires more than simply summing the properties of individual patches or ecosystems. It is crucial to understand the interactions between patches to facilitate the proper functioning of the landscape. Over more than 30 years, our analysis has demostrated a consistent loss (445.98 ha) and fragmentation (related to six spatial processes of landscape transformation), where disturbed patches have increased in number, decreased in size, and are closely related to the largest patch, RVSPV.

Our findings underscore the importance of these interactions, emphasizing the urgent need for targeted conservation efforts. While the landscape in Chorrillos has experienced significant loss and fragmentation, it has also shown increased heterogeneity, resulting in a more diverse array of land cover and habitats. Although these structural changes may benefit some species, others are severely affected. In the case of the RVSPV, some birds species have shown a degree of resilience by adapting to different types of land use and cover types, especially in the largest patch. However, these adaptations do not guarantee long-term survival in an increasingly fragmented habitat with declining habitat quality. Our results reflect broader trends in other urban wetlands in Lima and coastal cities such as those in Chile, highlighting the need for regional-level conservation strategies. We also recommend incorporating the perspectives of local stakeholders in future research to understand better the socioeconomic factors contributing to wetland degradation. This would complement the quantitative findings with a more holistic approach.

After identifying critical fragmentation areas with their spatial changes, such as size reduction and patch isolation, and their effects, including increased edge effects, we propose conducting more specific studies to analyze the impact of roads on Protected Natural Areas (PNAs). Such studies should address the impact of motor traffic on protected areas, considering factors like traffic intensity, density, proximity, light and acoustic pollution, and wildlife mortality caused by road accidents. Additionally, research on buffer zones in urban environments is also warranted. Enhancing the ecological function of patches and creating habitat links through spatial planning will benefit from a "stepping stone" approach.

This study advocates for further exploration in landscape ecology to enhance connectivity and ecological exchange between fragments, aiming to mitigate landscape loss. Additionally, a more detailed analysis of edge effects on the largest patch of the RVSPV is crucial, particularly considering abiotic, biological (direct and indirect) effects [50], among others, to develop specialized assessments of environmental quality. Given the current state of the RVSPV’s largest patch, we propose designing and implementing territorial restoration policies that recognize PNAs as fragile and fragmented spaces of high ecological/environmental value to avoid land-use conflicts in adjacent areas, particularly in coastal regions experiencing formal/informal urban growth in the city of Lima. Moreover, to protect migratory bird species that rely on the RVSPV’s largest patch as a refuge, it is necessary to develop mapping tools that take their movements when planning spatial policies [68] in the RVSPV area. Finally, we hope that this article will encourage more studies that will promote multi-species coexistence and support restoring resilient and adaptive spaces like urban wetlands.
